# Synovial Fibrosis Involvement in Osteoarthritis

**DOI:** 10.3389/fmed.2021.684389

**Published:** 2021-05-26

**Authors:** Li Zhang, Runlin Xing, Zhengquan Huang, Liang Ding, Li Zhang, Mingchao Li, Xiaochen Li, Peimin Wang, Jun Mao

**Affiliations:** ^1^Departments of Orthopedics, The Affiliated Hospital of Nanjing University of Chinese Medicine, Nanjing, China; ^2^Jiangsu Province Hospital of Chinese Medicine, Nanjing, China

**Keywords:** osteoarthritis, fibrosis, synovitis, fibroblast-like synoviocytes, extracellular matrix

## Abstract

Bone changes have always been the focus of research on osteoarthritis, but the number of studies on synovitis has increased only over the last 10 years. Our current understanding is that the mechanism of osteoarthritis involves all the tissues that make up the joints, including nerve sprouting, pannus formation, and extracellular matrix environmental changes in the synovium. These factors together determine synovial fibrosis and may be closely associated with the clinical symptoms of pain, hyperalgesia, and stiffness in osteoarthritis. In this review, we summarize the consensus of clinical work, the potential pathological mechanisms, the possible therapeutic targets, and the available therapeutic strategies for synovial fibrosis in osteoarthritis to gain insight and provide a foundation for further study.

## Rheumatology key Messages

Synovial fibrosis is closely associated with joint pain, hyperalgesia, and stiffness in osteoarthritis.

Sounder diagnostic criteria should be established for OA-related synovial fibrosis.

The mechanism of synovial fibrosis is being investigated, and available therapeutic strategies require further study.

## Introduction

Osteoarthritis (OA) is the most common degenerative joint disease and is characterized by pain, stiffness, and limited function in the clinic (1). In 2017, OA affected nearly 303 million people worldwide, including ~263 million people with knee OA and 40 million people with hip OA (2). Bone changes, such as the progressive loss and destruction of articular cartilage, thickening of the subchondral bone, and the formation of osteophytes, reflect the pathogenesis of OA, so the study of cartilage and subchondral bone in OA has always been a priority (3). Such studies are highly consistent with the etiology of OA, which involves aging, mechanical stress, and environmental changes in the joints. Bone changes may be the determining factor for the eventual use of surgical treatment for OA; unexpectedly, the consistency of the bone structure with clinical symptoms remains unclear, at least in terms of pain (4, 5). This implies that further efforts are needed to discover the pathological mechanisms of OA, especially those related to OA symptoms.

As OA involves chronic low-grade inflammation, the presence of an inflammatory microenvironment is likely to affect all tissues constituting the joint (6). It is widely accepted that synovitis can occur in the early stage of OA, promoting the development of OA throughout the whole pathologic process. Therefore, non-steroidal anti-inflammatory drugs (NSAIDs) are strongly recommended for clinical treatment, and OA is considered to be a highly prevalent rheumatic musculoskeletal disorder (7, 8). As cartilage destruction partly depends on the effect of inflammation, which disrupts the balance between synthesis and degradation in the extracellular matrix (ECM) (7), it may also be valuable to evaluate the damage caused by inflammation in the synovium. Overall, one major outcome of inflammation or inflammatory exudation is fibrosis, especially in the lung, liver, and kidney. In OA, synovial fibrosis (SF) is an imbalance caused by fibroblast proliferation and the disturbance of collagen synthesis and degradation, ultimately leading to excessive collagen deposition in the ECM (9, 10).

Recent research has also revealed that the ECM plays multiple roles in OA (11, 12). This may indicate that SF is not only a pathological outcome but also a likely pathogenic factor. SF is often accompanied by angiogenesis in both OA and rheumatoid arthritis (RA) (13). Recent studies have also found evidence for increased sensory innervation in the synovium in knee OA, but there is still no direct evidence on whether SF is associated with pain (14, 15). Does joint stiffness due to fibrosis associate with OA pain? Is SF associated with increased sensory innervation? Can the progression of SF be blocked when synovitis is alleviated? Obtaining a narrative review of SF in OA is an interesting research direction; thus, we searched PubMed with the keywords “fibrosis,” “OA,” and “RA.” We reviewed the pertinent literature to answer the following questions: What do we know about SF in osteoarthritis?

### Synovial Fibrosis and Synovitis

Synovitis is a typical chronic aseptic inflammation. Common symptoms caused by synovitis include pain, local temperature rise, swelling, joint movement limitation, and the severity of these symptoms is related to the degree of joint effusion (16). Synovitis is also known to produce a large number of pro-inflammatory factors, such as tumor necrosis factor (TNF), interleukin-1β (IL-1β), IL-6, IL-8, IL-15, IL-17, IL-21, inflammatory mediators, including PGE2, NO, adipokines, and matrix metalloproteinases (MMP-1, MMP-3, MMP-9, MMP-13), which lead to cartilage destruction, amplifying synovitis and ultimately creating a vicious cycle (17, 18). Besides, synovitis promotes the production of pain neurotransmitters, such as nerve growth factor and bradykinin (19). At the same time, synovitis promotes synovial angiogenesis, which in turn accelerates inflammation and leads to SF directly (20).

Usually, SF appears in the later stages of OA, which is different from synovitis. But in a study examining the effects of the intra-articular application of bupivacaine and levobupivacaine, inflammation and late fibrosis were found shortly after injection, suggesting that synovitis promotes fibrosis (21). On the other hand, as an aseptic chronic inflammatory disease, SF may be the inevitable outcome of “damage-repair,” and thus it can be emphasized that synovial inflammation drives the development of fibrosis. Notably, current studies cannot conclude that SF can cause synovitis independently, and whether synovial fibrosis can exist independently of synovitis, remains a topic of great interest to OA research. As for the relationship between synovitis and SF, maybe it is not well-understood what is the hen and what the egg, but this question is the one to inspire researchers' in-depth research.

### Synovial Fibrosis in Osteoarthritis

#### Clinical Status of Synovial Fibrosis

Much evidence has shown that SF is one of the most important causes of joint stiffness, synovial hyperplasia, and limited function, which are common symptoms in moderate and severe OA; other evidence also confirmed that a higher SF score is correlated with lower scores for KL grade, which indicates that SF may be negatively associated with clinical symptoms of OA (22, 23). This is because generalized pain is a major claim in OA patients, while independent joint stiffness does not occur very often. When joint stiffness begins to bother OA patients and joint movement is limited, loss of function becomes a reality.

Surgical treatments for OA, such as total knee arthroplasty (TKA), can cause arthrofibrosis, a fibrosing pathology of the synovial membrane, and the infrapatellar fat pad (24). In contrast to moderate and severe OA, TKA eliminates the effects on cartilage and the meniscus, so post-operative pain and dysfunction derived from SF can be observed more easily. Kalson et al. attempted to establish criteria for the diagnosis, classification, and severity grading of soft-tissue fibrosis after TKA and suggested that the diagnosis of fibrosis after TKA should be based on the exclusion of other causes of stiffness, the range of movement of the knee, the pathological anatomy and histopathology (Table 1) (25). These recommendations may also be adapted for the diagnosis of SF associated with primary OA.

**Table 1:** Criteria for the diagnosis, classification and severity grading of soft-tissue fibrosis after TKA established by Kalson et al.

	**Category**	**Criteria**	**Exclude**
Main diagnostic criteria	Restricted ROM	①Soft-tissue fibrosis that was not present preoperation. ②Loss of movement on extension>5°. ③Flexion range ≤ 100°	Problems with implant (malpositioning, cement, ectopic bone formation, loosening, malalignment); ligament reconstruction, infection, pain, CRPS or other specific causes; wound issues, incorrect surgical indication
Secondary diagnostic criteria	Stiffness		
	Pain		
	Inflammatory markers	CRP, WBCs	
	Aspiration of the joint	Microbiological culture and cell count	
Auxiliary diagnosis	X-ray, CT		Component malalignment; heterotopic ossification; patella infera
	MRI	Measurement of perisynovial thickness or quantification of fibrotic tissue in the parapatellar gutters	Focal fibroses; scar tissue
	Open or arthroscopic surgery	Direct visualization of fibrosis	
	Pathological anatomy and histopathology	Supply evidence of fibrosis, not essential; characterized by a varying degree of cellularity of fibroblasts.	

Recent developments in MRI and ultrasound have made it possible to investigate SF, but there is currently not enough evidence for routine use (26, 27). The degree of synovial thickening, not the volume, has been proven to be correlated with the level of SF on MRI, but others reported that the correlations between these factors were very weak (28, 29). This is likely because the MRI evaluation is based on the synovitis score, which fluctuates at different stages of OA, while the extent of fibrosis is relatively fixed. Ultrasound has also been shown to be useful in detecting and quantifying synovial abnormalities, especially for synovitis, as Doppler signals indicate active inflammation and vascularization in synovial arthritis but not fibrosis (30, 31). Laboratory tests of the synovium may be more advantageous for SF assessment than imaging evidence, and methods such as identification of cell phenotypes, quantitative detection of profibrotic markers, immunohistochemistry of collagen, and even HE staining can provide some guidance (32). According to the criteria established by Ruppert et al. for HE staining of sections, SF can be divided into three levels according to the ratio of the fibroblast-like synoviocyte (FLSs) length to the distance between FLSs (33). It seems that the “gold standard” evaluation for SF in OA is histology, although this requires an invasive biopsy that may not be applicable or acceptable to all patients.

#### Pathological Characteristics of Synovial Fibrosis

The normal synovium can be divided into the intima (synovial lining) and the subintima (outer layer). The intima comprises one to three layers of specialized columnar FLSs, which are interspersed with macrophages, while the subintima consists of multiple types of connective tissues, such as fibrous dense collagen, adipose tissue, or loose collagens. This layer is rich in type I and III collagen and microvascular blood supply, accompanied by lymphatic vessels and nerve fibers, but is relatively acellular (34, 35). From histological patterns, the synovium in OA patients is characterized by intima hyperplasia, subintima fibrosis, and stromal vascularization (36). In the latest report, scholars demonstrated increased innervation of the medial synovium after KOA surgical modeling, and the medial compartment of OA knees exhibited striking changes in Na_V_1.8^+^ innervation (7). Oehler et al. subtyped osteoarthritic synoviopathy and identified four patterns of OA-associated synoviopathy: hyperplastic, fibrotic, detritus-rich, and inflammatory synoviopathy (37). Interestingly, excluding hyperplastic synoviopathy, the remaining three subtypes are nearly all overlapping, with different emphases and degrees of inflammation. The fibrotic type has most of the characteristics of the other three types, but there is no macromolecular cartilage and bone debris, which is one of the typical features of detritus-rich cartilage. This indicates that OA induced by different factors could involve different subtypes of synoviopathy with varied features, and at least the fibrotic type originates more from inflammation than from cartilage debris. In summary, SF associated with OA should be identified as the accumulation of collagen under pathological conditions, dominated by abnormal remodeling of collagen types I and III in the subintima, together with angiogenesis and nerve invasion. Therefore, in the study of SF, angiogenesis and nerve invasion should also be considered.

The function of the normal synovium is mainly reflected by FLSs, as they are involved in the production of hyaluronan, collagens, and fibronectin in the intima and synovial fluid. This is essential for joint movement and cartilage nutrition (38). Macrophages make up a minority of cells in the normal intima, but their numbers increase dramatically in inflammatory arthritis (36, 38). Both types of cells are involved in SF associated with osteoarthritis. Surprisingly, these cells not only determine ECM changes but also dictate the functions of resident cells within tissues. The ECM supplies cells with proper chemical and mechanical signals to regulate cell proliferation, migration, and differentiation to maintain tissue homeostasis (36, 39). In SF, collagen I exhibit a disorganized structure and enhanced cross-linking, while collagen III is crucial for appropriate collagen I fibrillogenesis and tissue functionality (39). Petersen et al. believed that markers of type I or III collagen turnover may reflect the severity of synovitis and SF, which is highly correlated with OA pain sensitivity. Fragments of type I, II, and III collagens were then investigated in blood from OA patients compared with blood from control individuals, revealing increased degeneration of type I and II collagen and decreased degeneration of type III collagen, which was highly correlated with localized hyperalgesia in response to pressure stimulation (40). In conclusion, abnormal secretion of synovial cells forms the pathological basis of SF due to the inherent effects of the ECM as a pathogenic factor and biomechanical stimuli involved in OA. The subsequent collagen environment presents a fibrotic state with changes in synovial permeability and mechanical properties, which may cause pain and stiffness in OA joints.

### Potential Etiology of Synovial Fibrosis

**Figure 1:**
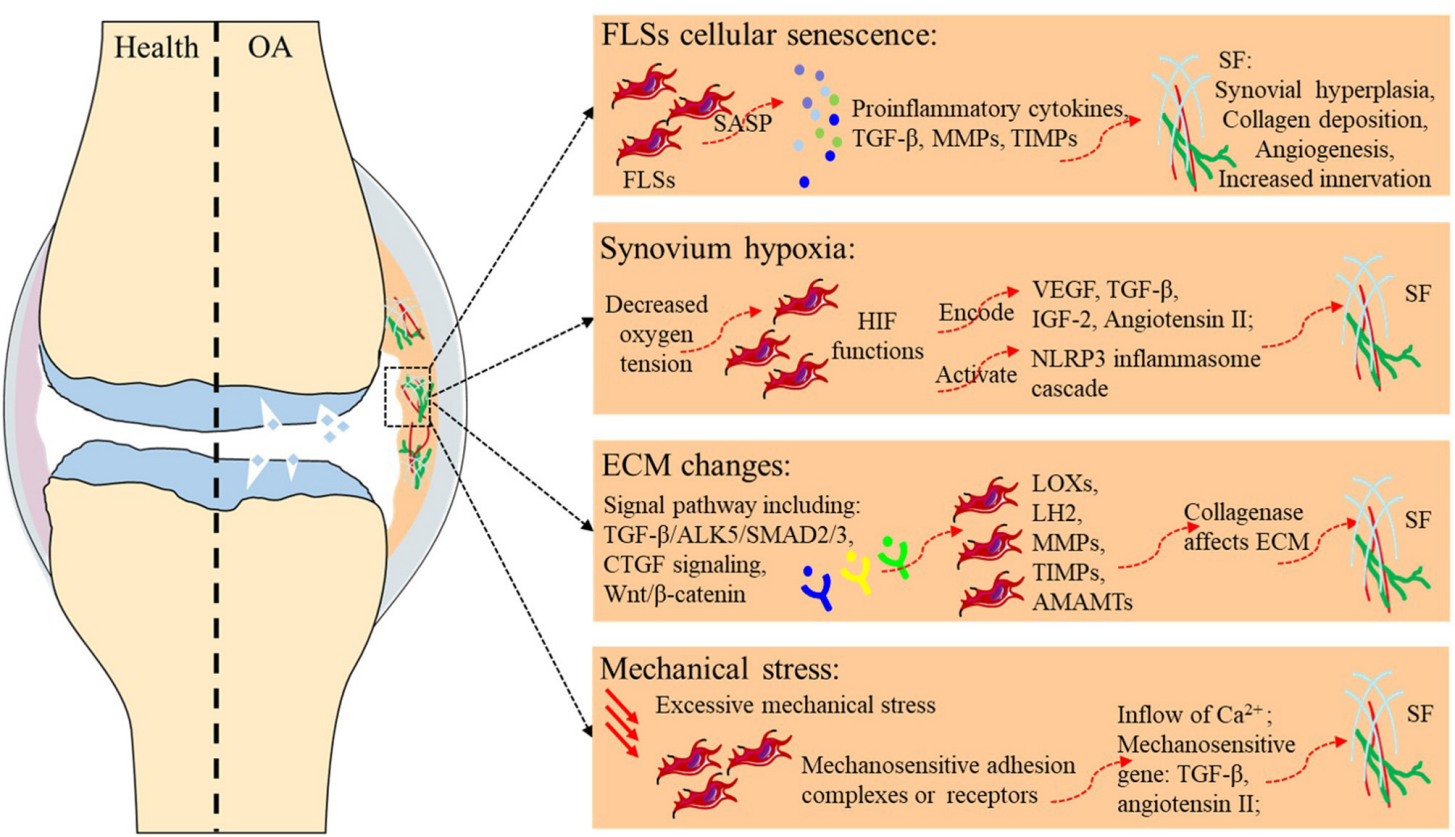
Potential etiology of synovial fibrosis in OA.

Fibrosis typically originates from abnormal tissue repair in response to wound healing. Fibroblasts activated by multiple diverse signals play a central role in this process, differentiate into myofibroblasts, and secrete matrix molecules to rebuild the ECM structure. Any risk factor for primary OA, such as aging, hypoxia, changes in the ECM environment, and mechanical stress, may play a similar role in SF (Table 2 and Figure 1). Although existing studies have not always targeted the synovium, evidence related to the balance of cartilage matrix degradation or fibrosis of other tissues can also provide some guidance for the study of SF.

**Table 2:** Fibrogenic factors in OA.

**Protein (encoding gene)**	**Risk factors**	**Function**	**Notes**	**References**
TGF-β and TGFβR	Aging	Receptors and ligands, signaling	Senescence-associated secretory phenotype	(41)
	Hypoxia		Positive feedback cycle between NLRP3 inflammasome activation and TGF-β1 induction	(42)
	ECM changes		Promotion of terminal differentiation of fibroblasts and the secretion of ECM components	(43)
	Mechanical stress		activation and release of TGF-β1	(44)
VEGF (*VEGF*)	Hypoxia	Growth factor	Modulated by HIF-1α at transcriptional level	(45)
IGF2 (*IGF*)	Hypoxia	Growth factor	Modulated by HIF-1α at transcriptional level	(45)
Angiotensin II	Hypoxia	Signaling	Modulated by HIF-2α at transcriptional level	(45)
NLRP3	Hypoxia	Signaling	Positive feedback cycle between NLRP3 inflammasome activation and TGF-β1 induction	(42)
IL-1β	Hypoxia	Cytokine	Increases TGF-β1 induction	(42)
LOXs and LOXL	ECM changes	Amine oxidases and LOX like proteins	Regulation of phosphorylation of Smad2/3 or p65 orERK1/2	(46–48)
LH2 (*PLOD2*)	ECM changes	Protease	PI3K/Akt signaling transduction; regulated by HIF or TGF	(49, 50)
CTGF	ECM changes	Growth factor	Reduction of Smad7 and promotion of TGF-β signaling	(51)

#### Aging

Directly relevant to aging is the study of cellular senescence, which refers to a state of cell cycle arrest, increased expression of cell cycle inhibitors, and enhanced production of proinflammatory cytokines, chemokines, and growth factors. A variety of stimuli and stresses, including telomere shortening, epigenetic changes, metabolic stresses, and mitochondrial dysfunction, can cause senescence. Markers for cellular senescence, including p16INK4A and p21, are upregulated in OA tissues, including cartilage, subchondral bone, and the synovium (41, 52, 53), suggesting cellular senescence in the FLSs of OA. Besides, senescence-associated secretory phenotype (SASP) is a pro-inflammatory secretory phenotype associated with cell senescence, including pro-inflammatory cytokines (such as IL-1α, IL-1β, IL-6, and IL-8), Tissue growth factors (TGF-β), MMPs, tissue inhibitors of metalloproteinases (TIMPs), and all these cytokines play important roles in SF. As cells in the synovium become proliferative and activated during SF, they may themselves become more susceptible to undergoing senescence. Thus, despite the lack of direct evidence, FLSs senescence is likely to promote the development of SF during aging.

#### Hypoxia

Hypoxia refers to a decrease in oxygen tension in tissues, and the central effector of the hypoxia response is the transcription factor hypoxia-inducible factor (HIF). In the hypoxic state, the alpha subunit in HIF is no longer hydroxylated but accumulates and translocates to the nucleus, where it binds to the beta subunit of HIF and exerts its function as a transcription factor (54). The genes encoding VEGF, TGF-β, and IGF-2, which are regulated by HIF-1α, and angiotensin II, which is modulated by HIF-2α, are all important profibrotic factors (45). Clinical studies have shown that HIF-1α levels in the serum, synovial fluid, and articular cartilage of knee OA patients are associated with progressive joint damage (55, 56). Hypoxic TGF-β1 induction increased succinate accumulation due to the reversal of succinate dehydrogenase activation and induced NLRP3 inflammasome activation in a manner dependent on HIF-1α induction. In response to NLRP3 inflammasome activation, the released IL-1β further increased TGF-β1 induction, suggesting the existence of a positive feedback cycle between inflammation and fibrosis in myofibroblast activation; this highlights the importance of studying SF associated with OA from the perspective of hypoxia (42).

#### Extracellular Matrix Changes

Remst et al. analyzed gene expression in TGF-β-stimulated human OA synovial fibroblasts and the synovium of mice with TGF-β-induced fibrosis, mice with experimental OA, and humans with end-stage OA. The genes encoding lysyl oxidase (LOX), pro-collagen-lysine, 2-oxoglutarate 5-dioxygenase 2 [PLOD2, also known as lysyl hydroxylase 2b (LH2b)], tissue inhibitor of metalloproteinase 1 (TIMP-1), collagen type I α1 chain (COL1A1), and collagen type V α1 chain (COL5A1) were upregulated under TGF-β stimulation, indicating that the signaling cascades of these key fibrotic factors were activated (57). Moreover, due to the vital role of matrix metalloproteinases, a disintegrin and metalloprotease (ADAMS) and a disintegrin and metalloproteinase with thrombospondin motif (ADAMTS) in ECM remodeling, the contributions of specific members in this family to SF should also be considered.

TGF-β plays a central role in the fibrotic cascade and is present as three isoforms (TGFβ1-3), all of which are elevated in OA patients and positively correlated with pain, loss of function, and radiographic staging (58). TGF-β signaling is initiated by binding to the TGF receptor, a heterodimer composed of TGFβR1 and TGFβR2. Further signal transduction is usually divided into SMAD-dependent classical pathways and non-classical pathways that are independent of SMAD. In the classical pathways, a phosphorylated TGFβR1, typically ALK5, can then transduce the TGF-β signal intracellularly to activate SMAD2/SMAD3, which complexes with SMAD4 to regulate gene expression. In contrast, the non-canonical pathway signals *via* other kinases, such as extracellular signal-regulated kinase, mitogen-activated protein kinase, nuclear factor-κB, and JUN amino-terminal kinase (43). TGF-β pathways promote the terminal differentiation of fibroblasts and the secretion of ECM components, especially collagen, fibronectin, and proteoglycans. A detailed description of the role of the TGF-β signaling pathway in OA is beyond the scope of this paper, but it is remarkable that TGF-β and its subfamily, bone morphogenetic proteins, play multiple roles in maintaining homeostasis of the cartilage and subchondral bone in OA. The TGF-β-mediated protective effects on cartilage matrix turnover rely not only on the production of ECM proteins such as type II collagen and aggrecan but also on the blockade of ECM protein degradation *via* increased production of protease inhibitors such as TIMP. Broeren et al. developed a 3-dimensional synovial membrane model involving micromasses made of either human primary synovial cell suspensions or a mixture of primary FLSs and CD14+ mononuclear cells. To recreate the synovial membrane in OA, the micromasses were exposed to TGF-β, which led to fibrosis-like changes in the membrane, including increased alpha smooth muscle actin (α-SMA) and increased expression of the fibrosis-related genes PLOD2 and COL1A1 (59). These results provide a detailed analysis of SF and show the suitability of this setup as a synovial membrane model for further research on RA and OA. Consistent with Broeren, Remst et al found that TGF-β induced PLOD2 expression in human FLSs *via* the ALK5/SMAD2/3 signaling pathway, thus aggravating SF in OA (60). In summary, high expression of TGF-β in the OA synovium accelerates OA progression, and inhibition of TGF-β in the synovium seems to be a favorable therapeutic strategy for SF. However, further research on TGF-β is still urgently needed due to its possible protective effects on cartilage.

The LOX family enzymes LOX and four lysyl oxidase-like proteins (LOXL1-4) are copper-dependent amine oxidases that catalyze the covalent cross-linking of collagen by oxidatively deaminating specific lysine and hydroxylysine residues in the telopeptide domains; this cross-linking increases collagen stiffness, which stiffens the ECM and promotes tissue fibrosis in the lung, myocardium, and liver. LOX may be induced by TGF-β1/Smad2/3 signaling, and knockdown of LOXL1 suppressed cell proliferation and fibrogenesis in TGF-β1-stimulated HSCs by regulating the phosphorylation of Smad2/3 (46, 47). Some research suggests that LOX expression was markedly elevated in OA-damaged regions of human cartilage and mouse OA cartilage induced by destabilization of the medial meniscus (DMM) surgery, and this elevated transcription caused cartilage destruction (61). Others have suggested that LOXL2 expression may be a protective response due to the inhibition of IL-1β-induced phospho-NF-κB/p65 and TGF-β1-induced ERK1/2 phosphorylation, although LOXL2 is upregulated in OA cartilage (48). These different results may be due to the varied expression of TGF-β and its receptors during different pathological stages of OA. Signal transduction in different environments may be a determinant of TGF-β and LOX function. Therefore, in the study of the OA synovium, researchers have observed that IL-1β simultaneously promotes LOX expression but has a depressing effect combined with TNF-α, while overexpressing LOX in the synovium exacerbates OA-related fibrosis (62, 63). Overall, LOX is closely related to tissue fibrosis through TGF signaling pathways, and the potential association with HIF-2α, mechanical conduction, and other OA-related factors may be a further direction for the study of SF.

PLOD2 encodes lysyl hydroxylase 2 (LH2), which catalyzes the hydroxylation of lysine intracellularly before the collagen is secreted. Then, LOX binds to hydroxylysine residues in the extracellular collagen fibers and induces cross-linking, the final step in the maturation of collagen, which is essential for the physical and mechanical properties of collagen fibrils (64). Aberrant lysyl hydroxylation and collagen cross-linking contribute to the progression of many collagen-related diseases, such as cancer and fibrosis. Wan et al demonstrated that PLOD2 expression was increased in endometrial carcinoma cells under hypoxic conditions and modulated the migration, invasion, and epithelial-mesenchymal transition of endometrial carcinoma cells *via* PI3K/Akt signaling (49). Other tumor diseases have also been reported to have similar pathological processes. In addition, PLOD2 is regulated by HIF-1 or TGF1 and mediates ECM remodeling, alignment, and mechanical properties through a transcriptionally mediated mechanism. Mia and Bank identified a selective inhibitor of IκB kinase, suppressed the expression of PLODs in dermal fibroblasts, and inhibited the TGFβ1-induced transition of fibroblasts into myofibroblasts, thus relieving excessive ECM synthesis (50). Gilkes et al. proved that HIF-1 activity in hypoxic fibroblasts promotes ECM remodeling by inducing the expression of the collagen hydroxylases P4HA1, P4HA2, and PLOD2 (65). In our most recent study, we explored the effect of inflammatory cascade amplification mediated by synovial macrophage pyroptosis on SF. High expression of TGF-β and PLOD2 in OA animals and FLSs was positively correlated with the degree of SF. Interestingly, TGFβ1, TGFβR1, LOX, PLOD1, and PLOD2 in the glenohumeral capsule of patients with shoulder instability may play a role in shoulder instability. We speculate that this correlation is closely related to SF of the shoulder joint, although the specific pathological mechanism still needs further study.

MMP1, which is also known as fibroblast collagenase, has mainly been implicated in mediating the degradation of type I collagen, which is most often mentioned in fibrosis as the major constituent of the fibrotic ECM. MMP1 cleaves collagen only between amino acids 775 and 776; thus, it is possible that hydroxylysylpyridinoline collagen cross-linked through aberrant PLOD2 and LOX is more difficult to degrade (66). It has been proven that ~0.1 Schiff base of LOX-mediated cross-linking per collagen molecule results in 2-3-fold higher resistance to human collagenase compared with that of un-cross-linked collagen (67). Therefore, despite the upregulation of both MMP1 and TIMP1 in the synovium in OA, the pathological changes of the synovial membrane continue to promote fibrosis, as indicated by not only the quantity of collagen but also the quality of collagen, as determined by its post-translational modifications, which actively drive the progression of fibrosis. In addition, MMP13 and ADAMTS-5 were also validated as drug targets that participate in the regulation of the ECM in OA, and ADAMTS-5 inhibitors were shown to reduce synovial joint damage in OA animal models.

Connective tissue growth factor (CTGF) is a well-known fibrogenic factor that has been shown to induce synovial fibrosis (60). It has been observed that both FLSs and chondrocytes were strongly induced to express CTGF after stimulation by TGF-β (60, 68). The main function of CTGF is to regulate proteoglycans on the cell surface, which can affect fibroblast proliferation, chemotaxis and accelerate ECM deposition (69). CTGF is thought to coordinate some fibrogenic effects through the TGF-β response element, but CTGF may also act independently of TGF-β (69, 70). Smad7, the inhibitory smad of TGF-β signaling, is reduced by CTGF, which in turn promotes TGF-β signaling, but the mechanism by which CTGF regulates Smad7 has not been fully elucidated (51). Therefore, it is valuable to further elucidate the induction effect of CTGF on synovial fibrosis in OA.

Wnt/β-catenin is closely associated with embryonic skeletal formation, tissue repair, fibrosis, and joint homeostasis (71). Wnt mediates several signaling cascades, especially the β-catenin–dependent (canonical) pathway (72), and β-catenin, as a transcriptional regulator, its stabilization or degradation is a central event in the Wnt signaling pathway. Existing studies show that the Wnt/β-catenin classical pathway has long been proven to be over-activated in the pathogenesis of OA (73). To be specific, increased expression of Wnt ligands and target genes was observed in both articular cartilage and synovium after injury, indicating Wnt signaling activation (74, 75). A recent study showed that XAV-939, a Wnt inhibitor, may reduce the proliferation of synovial fibroblasts and type I collagen levels by inhibiting the Wnt pathway, ultimately exerting a protective effect on synovial fibrosis (73). In conclusion, the Wnt/β-catenin signaling pathway may be a key molecular mechanism in the treatment of synovial fibrosis in OA, which may provide new ideas for the treatment of OA.

#### Mechanical Stress

Physical activity is one of the most frequently recommended non-pharmacological therapies for OA, but the duration and intensity of exercise vary widely. Moderate mechanical stress may reduce sensitization to the inflammatory response in the articular cartilage and chondrocytes and be beneficial for OA (76). However, excessive mechanical stress exacerbates OA progression by inducing chondrocyte apoptosis and osteophyte formation (77, 78). This indicates that mechanical stimulation can regulate the balance of synthesis-degradation in cartilage and osteogenesis-osteoclastogenesis in the subchondral bone. *In vivo*, mechanical stress is transduced into the cell from the sites at which the cells attach to the ECM. The cells may engage their ECM both *via* mechanosensitive adhesion complexes and *via* other surface receptors, including those for growth factors and inflammatory mediators, which cannot act as adhesive anchors but may modify the mechanical signals transduced at the cell/ECM interface (79). Under these conditions, activated mechanosensitive plasma membrane channels allow the inflow of Ca^2+^ that can act as a second messenger to regulate gene expression. TGF-β1 is a typical mechanosensitive gene, and previous studies have suggested that mechanical stretching activates and releases latent TGF-β1 in living tissues from fibrotic lungs (44). In cardiac fibrosis, mechanical stress is a major factor for cardiac hypertrophy in response to pressure or volume overload, and angiotensin II seems to be another mechanosensitive gene that promotes fibrosis (80). As the FLS response to mechanical stress is critical during the initial stages of OA, SF caused by excessive mechanical stimulation is likely to occur (81), and subsequent ECM stiffness may affect tissue delivery of mechanical signals and exacerbate OA progression.

### Existing and Potential Treatments for Synovial Fibrosis

Open surgery for SF is undoubtedly the most direct and effective treatment, but it often requires large incisions with extensive exploration of the joint and surrounding extracapsular soft tissues. Unfortunately, the surgery itself induces a fibrotic process, and the outcomes of post-traumatic surgery are poor, with most patients unable to return to their presurgery level of function. Even if satisfactory results are achieved during the operation, SF is likely to recur within a certain period.

Recently, SF research has made some progress in conservative drug treatment. Numerous studies have consistently reported that PRG4 and HA attenuate profibrotic responses to TGF-β in OA animals or FLSs. Interestingly, FLSs themselves can synthesize and secrete PRG4 and HA. Correcting the pathological state of FLSs in OA seems to be of great significance for the treatment of SF. In this context, Qadri et al concluded that increasing intracellular cAMP levels in FLSs mitigates SF through enhanced production of HA and PRG4 (82). Plaas et al. proved that HA injection blocked all gait changes and protected joints from femoral cartilage erosion and tissue fibrosis in KOA mice, and they deduced that HA injection could mimic the protective effects of ADAMTS-5 ablation (83). Their further findings supported this hypothesis and demonstrated that ADAMTS-5 was blocked by a CD44-dependent mechanism (84). As PRG4 is a ligand of the CD44 receptor, Qadri et al examined the role of the PRG4-CD44 interaction in regulating SF in OA and demonstrated that PRG4 inhibited fibroblast-to-myofibroblast transition, thus downregulating the expression of fibrotic genes in the OA synovium (85).

Furthermore, regarding the balance between MMP and TIMP expression in FLSs from KOA with flexion contracture using adenovirus-mediated relaxin gene therapy, relaxin could serve as an alternative therapeutic agent during the initial stage of OA with flexion contracture by exerting antifibrogenic effects (86). In addition, methylene blue, NSAIDs, and salmon calcitonin were also reported to have therapeutic effects on SF, although their specific mechanisms are still unclear (87–89). The Wnt/β-catenin signaling pathway and senescent cells are potential targets for antifibrosis therapy, but the intervention procedure needs further exploration (73, 90).

## Conclusion

Evidence from direct research on SF in OA and related studies suggests the following. (I) Fibrosis is the outcome of inflammation. It is not clear whether the ongoing process of SF has a pathogenic effect in OA, especially in relation to pain. If so, effective intervention to slow the progression of fibrosis is necessary even if fibrosis is irreversible, as the greatest advantage is the improvement of joint function and the relief of OA symptoms. (II) Although we have some understanding of ECM environmental changes and the mechanism by which they are involved in the pathological process of SF associated with OA, our knowledge of this pathological mechanism is still insufficient. Angiogenesis and nerve invasion are likely to have a strong correlation with the pathological process of SF and may be involved in the development of SF, which deserves further exploration. (III) Existing research on the pharmacodynamic targets and intervention effects of SF is not sufficient, and further exploration is still needed in the future. Notably, the combined use of antifibrotic drugs has high potential during anti-inflammatory therapy for OA.

## Author Contributions

PW and JM conceptualized the current study. LZ (1st author) and RX drafted and revised the paper. ZH, LD, and LZ (5th author) provided the relevant literature. XL and ML were responsible for the proofreading. PW provided final approval of the version to be submitted. All authors contributed to the article and approved the submitted version.

## Conflict of Interest

The authors declare that the research was conducted in the absence of any commercial or financial relationships that could be construed as a potential conflict of interest.
